# Endoscopic ultrasound-guided treatment for non-stenotic cholangitis in transplant recipient with hepaticojejunal anastomosis

**DOI:** 10.1055/a-2818-8468

**Published:** 2026-03-11

**Authors:** Margherita Pizzicannella, Dario Ligresti, Gabriele Rancatore, Dario Quintini, Maria Vittoria Grassini, Giacomo Emanuele Maria Rizzo, Ilaria Tarantino

**Affiliations:** 1 18326 Endoscopy Unit, IRCCS-ISMETT, Palermo, Italy

Cholangitis following orthotopic liver transplantation (OLT) is commonly associated with biliary obstruction, such as anastomotic strictures; however, rarely, it may develop despite preserved biliary drainage. In patients undergoing OLT with Roux-en-Y hepaticojejunostomy (HJ), non-stenotic cholangitis (NSC) represents a distinct and poorly understood clinical entity. Its pathogenesis is likely multifactorial and has been attributed to the reflux of enteric contents into the biliary tree through the Roux-en-Y limb, chronic inflammatory changes of the bile ducts, and altered cholangiocyte membrane functions 1 . NSC significantly compromises the patient’s quality of life through repetitive febrile episodes, recurrent hospitalization, prolonged exposure to antibiotic therapy and, in the long term, potentially secondary sclerosing cholangitis with liver fibrosis and cirrhosis 2 .

The management of NSC is challenging consisting of targeted antibiotic therapy and revisional surgery in selected cases with a high rate of recurrence 3 .

We report the case of a 71-year-old man who underwent OLT with Roux-en-Y HJ for hepatocellular carcinoma arising in hepatitis B virus–related liver cirrhosis ( Video 1 ). Several years after surgery, the patient developed recurrent episodes of cholangitis. Magnetic resonance imaging demonstrated dilatation of the intrahepatic and extrahepatic biliary ducts without evidence of an anastomotic stricture. Delayed-phase images showed reflux of the contrast medium into the non-functional loop, with subsequent retrograde opacification of the intrahepatic biliary ducts, supporting a diagnosis of non-stenotic cholangitis.

EUS-guided choledochoduodenal anastomosis was performed to treat non-stenotic cholangitis in a patient with Roux-en-y HJ after orthotopic liver transplantation. EUS, endoscopic ultrasound; HJ, hepaticojejunostomy.Video 1

**Fig. 1:**
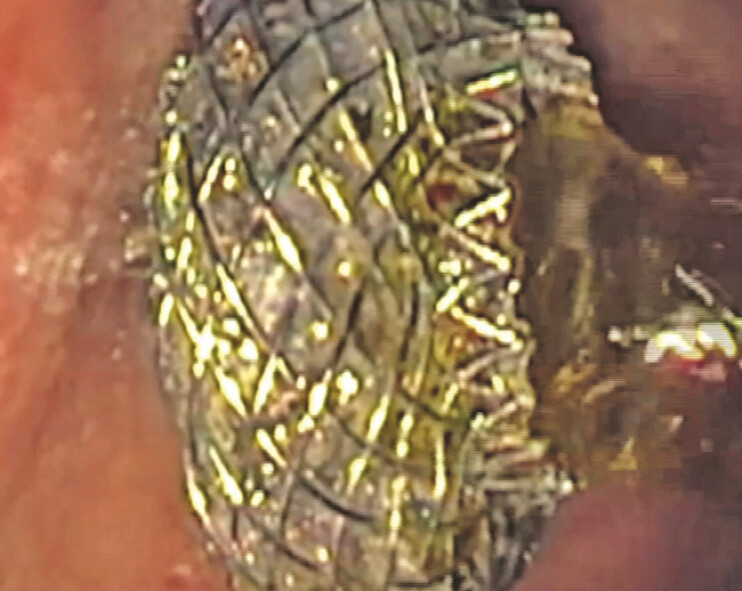
Bile flowing out of the distal flange of the lumen apposing metal stent, correctly positioned in the duodenal bulb.

**Fig. 2:**
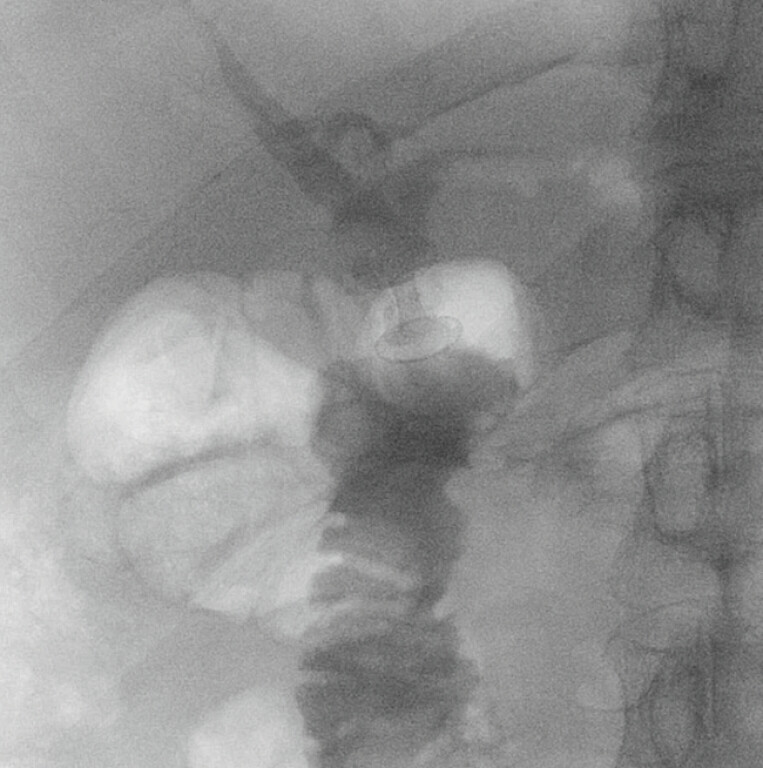
A fluoroscopic image showing the passage of the contrast medium through the stent in the biliary tree and duodenum, confirming the correct positioning of the stent at the end of the procedure.

**Fig. 3:**
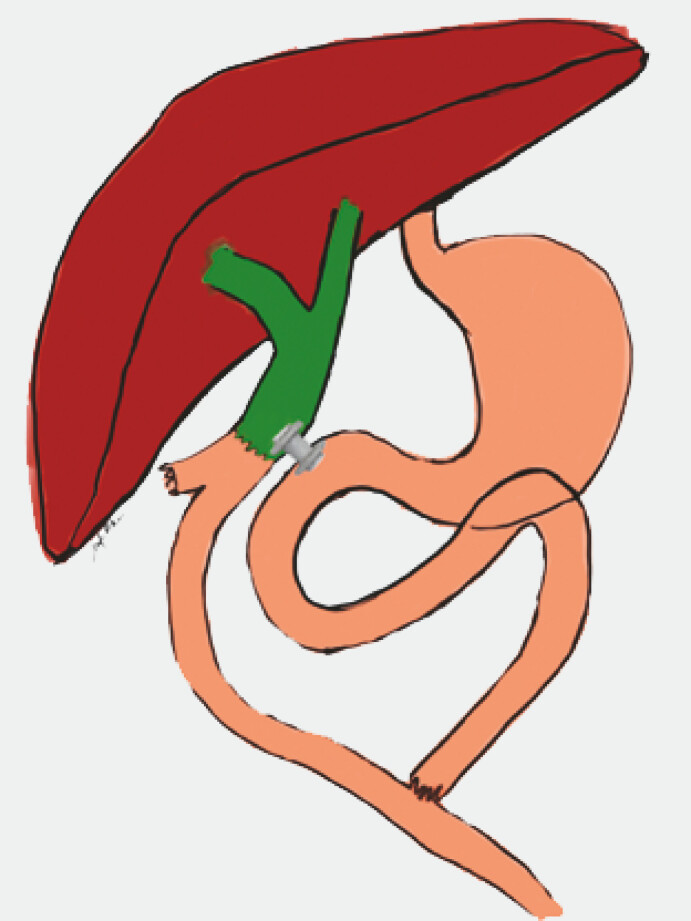
Schematic illustration showing a lumen-apposing metal stent positioned between the duodenal bulb and the common bile duct above the hepaticojejunal anastomosis. The stent facilitates bile flow into the duodenal lumen, thereby preventing reflux within the biliary tree.

Endoscopic ultrasound-guided choledochoduodenostomy was performed under general anesthesia by placing an 8 × 8 mm lumen-apposing metal stent between the common bile duct and the duodenal bulb ( Fig. 1 , Fig. 2 , Fig. 3 ). The procedure restored antegrade bile flow directly into the duodenum, resulting in interruption of loop related stasis and biliary reflux. The post-procedural course was uneventful. An oral liquid intake was resumed on the day following the procedure, and the patient was discharged on postoperative day 2. During a 6-month follow-up period, no further episodes of cholangitis were observed.

Endoscopic ultrasound (EUS)-guided biliary drainage of the residual dilated biliary tract allowed the resolution of NSC in a frail patient with previous surgical HJ. EUS-guided interventions represent a promising mini-invasive therapeutic option, alternative to surgery, with expanding indications, including selected benign biliary conditions.

Endoscopy_UCTN_Code_CCL_1AZ_2AN Endoscopy_UCTN_Code_TTT_1AS_2AH
